# Retrogradation enthalpy does not always reflect the retrogradation behavior of gelatinized starch

**DOI:** 10.1038/srep20965

**Published:** 2016-02-10

**Authors:** Shujun Wang, Caili Li, Xiu Zhang, Les Copeland, Shuo Wang

**Affiliations:** 1 Key Laboratory of Food Nutrition and Safety, Ministry of Education, College of Food Engineering and Biotechnology, Tianjin University of Science & Technology, Tianjin 300457, China; 2 Faculty of Agriculture and Environment, The University of Sydney, NSW 2006, Australia.

## Abstract

Starch retrogradation is a term used to define the process in which gelatinized starch undergoes a disorder-to-order transition. A thorough understanding of starch retrogradation behavior plays an important role in maintaining the quality of starchy foods during storage. By means of DSC, we have demonstrated for the first time that at low water contents, the enthalpy change of retrograded starch is higher than that of native starch. In terms of FTIR and Raman spectroscopic results, we showed that the molecular order of reheated retrograded starch samples is lower than that of DSC gelatinized starch. These findings have led us to conclude that enthalpy change of retrograded starch at low water contents involves the melting of recrystallized starch during storage and residual starch crystallites after DSC gelatinization, and that the endothermic transition of retrograded starch gels at low water contents does not fully represent the retrogradation behavior of starch. Very low or high water contents do not favor the occurrence of starch retrogradation.

Rice and wheat are two of the most economically important crops, contributing food for more than 90% of the world’s population^1^. The quality of food products made from these staple grains is largely dependent on the properties of their major component starch, which is the most abundant storage carbohydrate in plants. Starch is used widely in food and non-food industries and, due to its low cost and renewability, it is also used to fabricate biodegradable materials. Before consumption by humans or for its application in materials, starch is usually gelatinized, by undergoing an order-to-disorder transition brought about by heating in the presence of water. On storage after gelatinization, the disordered starch chains gradually reassociate into a different ordered structure in a process termed retrogradation. Starch retrogradation is accompanied by a series of physical changes such as increased viscosity and turbidity of pastes, gel formation, exudation of water and increased degree of crystallinity with the appearance of B-type crystalline polymorphs^2^. The changes that starch undergoes during gelatinization and retrogradation are irreversible and are major determinants of its functional properties for food processing, during digestion, and in industrial applications. These properties determine the quality, acceptability, nutritional value, and shelf-life of finished, starch-rich foods^3^.

Gelatinization of starch causes loss of molecular order in both the amylose and amylopectin components. Subsequent retrogradation is the process in which the disaggregated starch chains reassociate into an ordered structure, which is different from the initial state^4^. Some of the partial crystallinity of amylopectin in native starch is restored during retrogradation^5^. Starch gelatinization and retrogradation are often characterized by differential scanning calorimentry (DSC), in combination with other structural or functional techniques^3^,^6^. The amount of water in the DSC pans plays an important role in the extent of starch gelatinization, which leads to the differential effects on subsequent retrogradation behavior. The effects of water availability on starch gelatinization have been investigated extensively, as reviewed recently by Wang and Copeland^3^. At low water content, the DSC endothermic transition only represents the limited swelling or incomplete gelatinization of starch granules^7^,^8^. Even at high water content (water:starch = 1.5:1 or higher), the DSC endotherm does not always represent the complete gelatinization of granules, as shown in studies with cereal and legume starches^7^,^8^,^9^,^10^. In comparison to gelatinization, the effect of water content on starch retrogradation has attracted much less attention^6^. A comprehensive study on starch retrogradation over a wide range of water content is lacking despite its major significance for the quality of foods during storage^6^. As most food systems are processed under limited water conditions^5^ and then stored at 4 °C, an understanding of the changes that starch undergoes during gelatinization and retrogradation in such conditions is important to manipulate the textural and nutritional qualities of starchy foods. Under water-limited conditions, starch undergoes only partial gelatinization on DSC heating and will retrograde in a different way during subsequent storage compared with fully gelatinized starch^8^.

Starch retrogradation may be characterized by determining the enthalpy change of retrograded starch gels after storage at 4 °C. The degree of retrogradation (DR) is often described by a parameter obtained by dividing the gelatinization enthalpy change of starch granules by the enthalpy change of reheated retrograded starch gels^11^,^12^. In the present study, the retrogradation behavior of wheat and rice starches over a wide range of water content was examined. To the best of our knowledge, this is the first study to investigate starch retrogradation over a wide range of water content by DSC in combination with ATR-FTIR and Raman spectroscopy. This study is aimed at gaining a better understanding of the effect of water content on starch retrogradation behavior in food and non-food applications.

## Experimental Section

### Materials

The wheat (Zhoumai 18) flour was kindly provided by the Institute of Crop Science, Chinese Academy of Agricultural Science. The rice (*Oryza sativa*) sample was purchased from a supermarket in Tianjin, China. Amylose (A0512) and amylopectin (A8515) from potato starch were purchased from Sigma Chemical Co. (St. Louis, MO, USA). Other chemical reagents were of all analytical grade.

### Isolation of Starch

Rice starch (RS) was isolated according to the method of Spigno and De Faveri^13^, and wheat starch (WS) according to a dough ball method^8^,^14^.

### Chemical Analysis of Starch

The amount of damaged starch in the isolated starches was determined using the Megazyme Starch Damage Kit (Megazyme International Ireland Ltd. (Bray Co., Wicklow, Ireland). Amylose content was determined by iodine binding according to Chrastil^15^ using a standard curve of 10%, 20%, 25%, 30%, and 35% potato amylose mixed with potato amylopectin. The crude lipid content of starch granules was determined gravimetrically by Soxhlet extraction using petroleum ether. The nitrogen content of wheat grains and starch granules were determined by standard Kjeldahl methodology. Crude protein content was estimated by multiplying the nitrogen content by a conversion factor of 6.25. Moisture content was determined by drying to constant weight at 105 °C and ash content was determined using a muffle furnace at 550 °C.

### Differential Scanning Calorimetry

Differential scanning calorimetry (DSC) measurements were performed using a Differential Scanning Calorimeter (200 F3, Netzsch, Germany) equipped with a thermal analysis data station. Starch (approximately 3 mg wet weight) was weighed accurately into an aluminum sample pan. Distilled water was added with a pipette to obtain starch:water ratios of 1:0.5, 1:0.75, 1:1, 1:1.5, 1:2.5 and 1:4 (w/v) in the DSC pans, corresponding to the water content of 33, 43, 50, 60, 71 and 80%, respectively. The pans were sealed and allowed to stand overnight at room temperature before analysis. The pans were heated from 20 to 100 °C at a rate of 10 °C/min. An empty aluminum pan was used as the reference. Starch retrogradation was determined on the same gelatinized samples after storage at 4 °C for 7 days. The retrograded starch samples were re-scanned using the heating profiles described for starch gelatinization. Gelatinization enthalpy change of native starch (ΔH_G_) and enthalpy change on reheating of retrograded starch gels (ΔH_R_) were obtained using data recording software. All measurements were performed in triplicate. Degree of retrogradation (%DR) was calculated according to the formula:


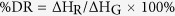


After DSC measurements, the sample pans were cooled to room temperature and reweighed. Those pans without any weight loss were collected and starch samples were freeze-dried and used for Fourier-transform infra-red (FTIR) and Raman spectroscopic analysis.

To obtain sufficient material to gain a better understanding of starch retrogradation behavior, retrograded starches were also prepared by simulating the DSC heating conditions without stirring using the RVA to prepare starch gels followed by storage at 4 °C for 7 days. The retrograded starch samples prepared in this way were freeze-dried, ground using a mortar and pestle, and passed through a 100 μm sieve. The resulting retrograded starch powders were mixed with water in ratios of 1:0.5, 1:0.75, 1:1, and 1:1.5 (w/v) in the DSC pans and analyzed using the same procedures for DSC measurements as described above.

### Attenuated Total Reflectance (ATR)-FTIR Spectroscopy

The residual molecular order of native starch after DSC heating and of retrograded starch gels after DSC reheating was determined directly using a Thermo Scientific Nicolet IS50 spectrometer (Thermo Fisher Scientific, USA). The ratio of absorbance at 1047/1022 cm^−1^ was used to estimate the short-range molecular order of starch.

### Laser Confocal Micro-Raman (LCM-Raman) Spectroscopy

The molecular order of native starch after DSC heating and of retrograded starch gels after DSC reheating was also determined by using a Renishaw Invia Raman microscope system (Renishaw, Gloucestershire, United Kingdom), which was equipped with a Leica microscope (Leica Biosystems, Wetzlar, Germany) and a 785 nm green diode laser source. Spectra were collected on at least five different spots of gelatinized and retrograded starch samples in the range of 3200–100 cm^−1^, with a resolution of approximately 7 cm^−1^. The full width at half height (FWHH) of the band at 480 cm^−1^, which can be used to characterize the molecular order of starch^6^, was calculated using the WiRE 2.0 software.

### Statistical Analysis

Results are reported as the mean values and standard deviations of at least duplicate measurements. Analyses of variance (ANOVA) by Duncan’s test (p < 0.05) were conducted using the SPSS 17.0 Statistical Software Program (SPSS Inc. Chicago, IL, USA).

## Results and Discussion

### Basic Composition of Rice and Wheat starches

Amylose contents of the rice and wheat starches were 11.5% and 25.0%, respectively (Table 1). The amylose content of starch from non-waxy rice varieties varies between 7% and 33%^16^,^17^,^18^, whereas non-waxy wheat starch usually contains 20–30% amylose^19^. Damaged starch contents of the rice and wheat starches were 0.89% and 1.30%, respectively, whereas ash, protein, lipid and water contents of the two starches were all within the range of values reported in the literature^20^.

### Thermal Properties of Native and Retrograded Starches

**Figure 1:**
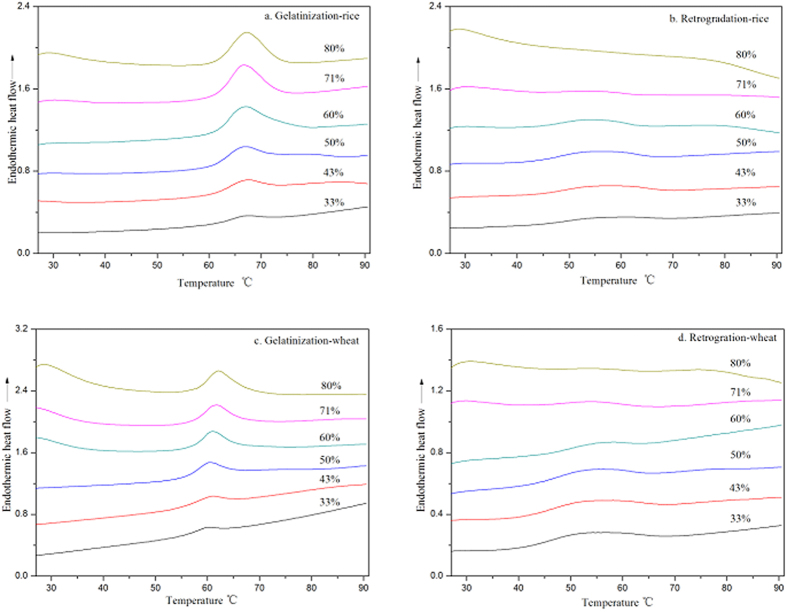
DSC curves of RS gelatinization (**a**), RS retrogradation (**b**), WS gelatinization (**c**), WS retrogradation (**d**).

The DSC curves of starch gelatinization and from reheated retrograded starch gels are presented in Fig. 1. Rice and wheat starches displayed a typical gelatinization endothermic transition in the temperature ranges of 61.2–73.9 °C and 55.1–67.4 °C, respectively. With increasing water content, the gelatinization endothermic transition became progressively more pronounced and symmetrical (Fig. 1a,c). The area of this gelatinization endotherm increased with increasing water content. On reheating the retrograded starch, much broader but shallower endothermic transitions ranging from 42.9–68.9 °C and from 41.9–65.3 °C were noted for rice and wheat starch, respectively (Fig. 1b,d). The area of this retrogradation endotherm increased initially and then decreased with increasing water content.

Thermal transition temperatures of native starch and retrograded starch gels at different water contents are listed in Table 2. There were small differences in the gelatinization transition temperatures of rice starch as water content increased from 31% to 80%. The T_o_, T_p_ and T_c_ of rice starch were in the range of 61.2–62.1 °C, 66.9–68.3 °C and 71.0–73.9°, respectively. The transition temperature range (T_c_–T_o_) was from 9.8 to 11.7 °C. The T_o_, T_p_ and T_c_ of wheat starch ranged from 55.1, 60.8 and 64.6 °C to 56.8, 62.1 and 67.4 °C, respectively. The thermal transition broadened with T_c_–T_o_ ranging from 9.5 to 11.3 °C. These observations are consistent with previous results, which showed that the conclusion temperature of pea and wheat starches increased gradually with increasing water content^7^,^8^.

After storage for 7 days, the transition temperatures of reheated retrograded starch gels, especially T_o_ and T_p_, were much lower than those of native starch granules (Table 2), indicating that the melting of crystallites in retrograded starch gels occurred more readily than for native starch crystallites. The T_o_ of retrograded starch gels did not vary greatly with different water content, suggesting similarities in the onset of melting behavior of crystallites in retrograded starch gels. However, the T_p_ and T_c_ of retrograded rice starch gels decreased from 56.8 to 53.9 °C and from 67.8 to 62.5 °C, respectively, as the water content increased from 33 to 71%. Similarly, the T_p_ and T_c_ of retrograded wheat starch gels decreased from 58.3 to 53.6 °C and from 65.3 to 62.7 °C, respectively. With increasing water content, T_c_–T_o_ decreased from 25.4 and 22.9 °C to 18.3 and 17.3 °C for rice and wheat starch, respectively.

Gelatinization enthalpy change increased from 2.6 to 14.4 J/g and from 2.9 to 10.7 J/g for rice and wheat starch, respectively, as the water content increased from 33 to 71%, above which the enthalpy change remained essentially the same (Table 3). This observation was consistent with previous results^8^. Rice starch presented a higher maximum enthalpy change of 14.4 J/g than did wheat starch (10.7 J/g). The maximum enthalpy change of native starch granules has been shown to be variety dependent^8^.

After retrogradation, the enthalpy change of rice and wheat starch gels varied according to water content (Table 3). Enthalpy change of rice starch gels increased from 3.9 J/g at a water content of 33% to a maximum value of 7.0 J/g at a water content of 50%, and then decreased to zero at a water content of 80%. In comparison, the retrogradation enthalpy change of wheat starch gels reached a maximum value of 6.2 J/g at a water content of 33%, and then decreased progressively to 0.5 J/g at a water content of 80%. When the water content was above 80%, no retrogradation of wheat starch gel was observed by DSC (data not shown), consistent with previous reports^21^,^22^,^23^.

**Figure 2:**
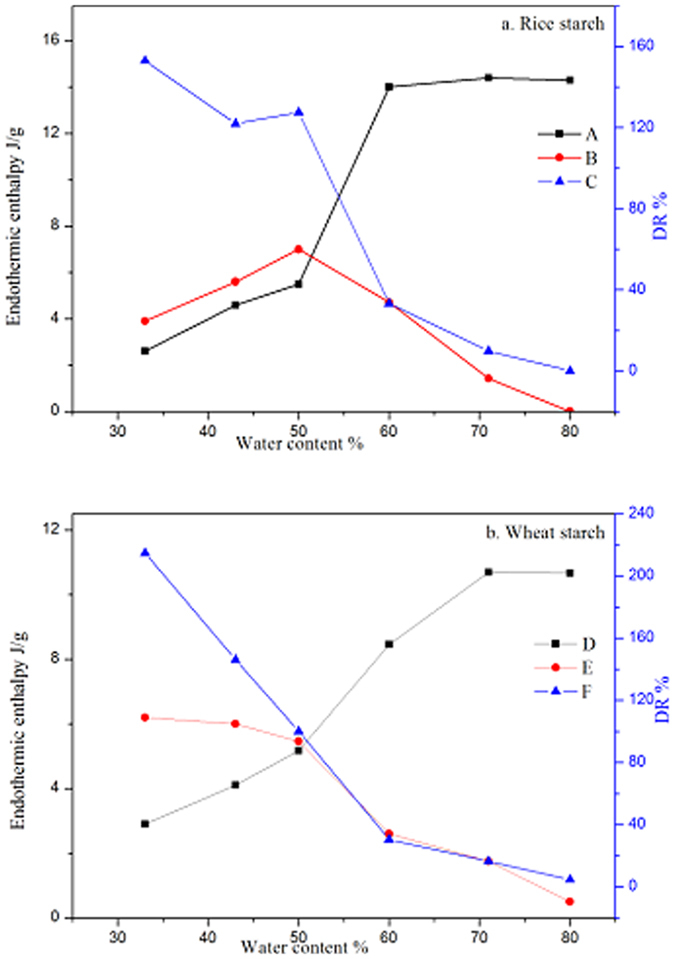
Gelatinization enthalpy change of RS (**A**), retrogradation enthalpy change of RS (**B**), DR of RS (**C**), Gelatinization enthalpy change of WS (**D**), retrogradation enthalpy change of WS (**E**), DR of WS (**F**).

Interestingly, the enthalpy change of retrograded starch gels was greater than that of native starch granules when the water content was between 33 and 50% (Table 3), resulting in a calculated value for the degree of retrogradation of the starch gels greater than 100% (Table 3, Fig. 2). This suggests that the broad endothermic transition of retrograded starch gels involves not only the melting of recrystallized starch formed during retrogradation, but the further melting of residual crystallites that remained in the retrograded starch gels. As proposed^8^, the DSC endothermic transition does not represent the full gelatinization of starch granules at DSC water-limited conditions.

**Figure 3:**
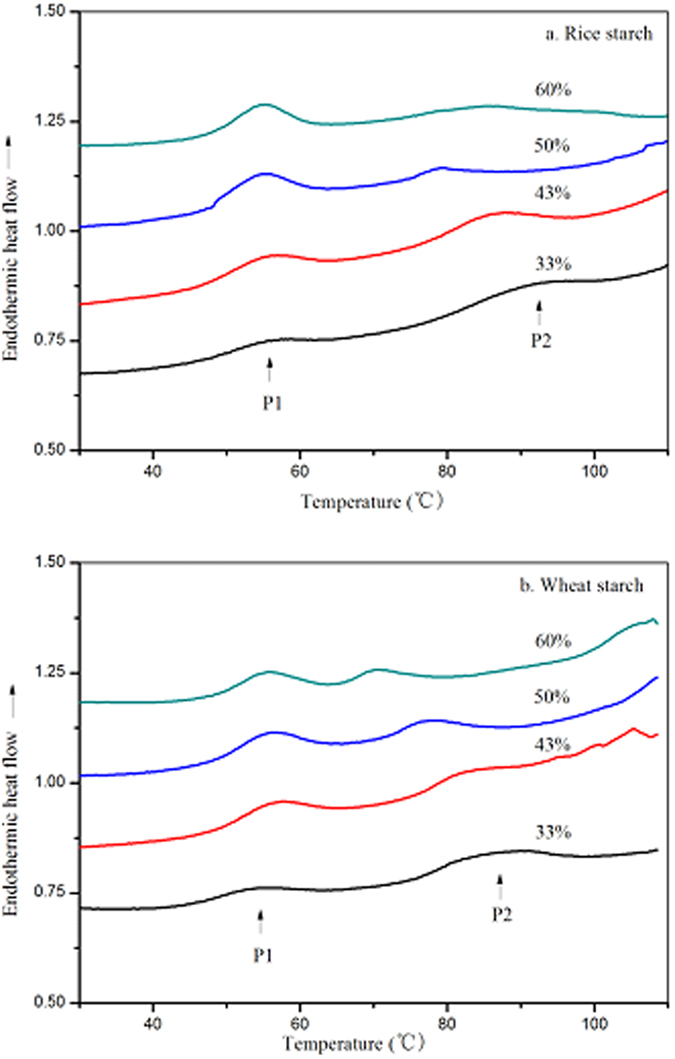
DSC curves of retrograded rice starch powders (**a**), retrograded wheat starch powders (**b**). p1: represents the melting of recrystallized starch during retrogradation; p2: represents the melting of residual starch crystallites after DSC gelatinization.

To further substantiate the above hypothesis, the thermal transition of freeze-dried retrograded starch powders was determined (Fig. 3). Two separated endothermic transitions were noted in the range of 40~65 °C and 70~100 °C. The lower temperature endotherms are proposed to be the melting of retrograded starch crystallites, whereas the ones at higher temperature are proposed to be due to the melting of residual crystallites remaining after DSC gelatinization. The observation of two separated endotherms indicated that melting of recrystallized starch formed during retrogradation and residual crystallites after DSC gelatinization occurred separately rather than simultaneously. The second endothermic transition is unlikely to be due to the melting of amylose-lipid complexes, which occurs at higher temperatures. Moreover, the lipid content of the starches was very low. The broad endothermic transitions noted for retrograded starch gels are consistent with the consecutive melting of recrystallized starch and residual starch crystallites. However, unlike the freeze-dried material, the endotherms of the reheated starch gels in the DSC pans may overlap because water is likely to migrate more readily from recrystallized starch to residual starch crystallites in gels than in rehydrated powders. Interestingly, the enthalpy change of freeze-dried retrograded starch powers was lower than that of retrograded starch gels (Table 3), consistent with the findings that freeze drying can disrupt the crystalline and molecular order of starch^24^. At higher water content of 60–80%, the degree of retrogradation was between 0–32.3% and between 4.7–34.1% for rice and wheat starch, respectively.

### Short-range Molecular Order of Starch by ATR-FTIR and Raman Spectroscopy

**Figure 4:**
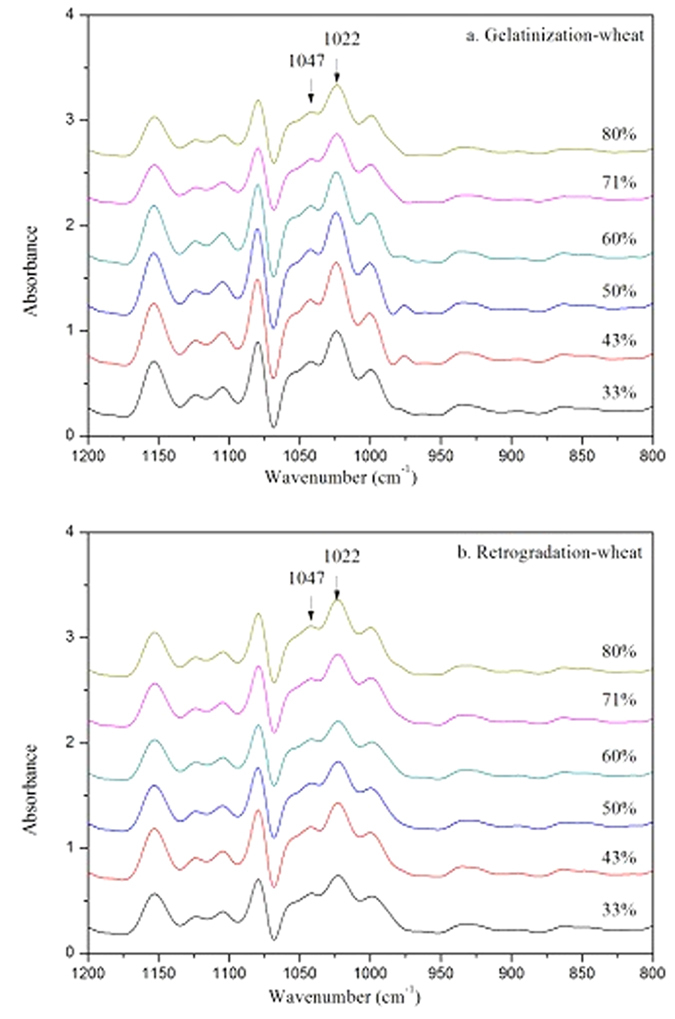
The deconvoluted FTIR spectra of gelatinized WS after DSC heating (**a**), reheated retrograded WS (**b**).

The short-range molecular order of double helices in starch can be characterized by the ratio of absorbances at 1047/1022 cm^−1^ obtained from FTIR spectroscopy of starch^25^,^26^,^27^,^28^. To verify the further melting of starch granule crystallites remaining in retrograded starch on DSC reheating, the molecular order of native starch after DSC heating and retrograded starch gels after DSC reheating was determined by ATR-FTIR and Raman spectroscopy. To identify the differences in the molecular order of these starch samples, the deconvoluted FTIR spectra of wheat starch in the range of 1200–800 cm^−1^ were obtained (Fig. 4). The ratios of 1047/1022 cm^−1^ of starch after gelatinization decreased with increasing water content (Table 4), indicating that the degree of disruption of molecular order in starch after DSC heating increased with increasing water content. Similar results were also observed with retrograded starch gels after DSC reheating, although in some cases no significant differences were observed. The ratios of 1047/1022 cm^−1^_R_ were lower than those of 1047/1022 cm^−1^_G_ over the whole range of water content, although the difference was small in some cases. This result showed that the molecular order of the retrograded starch gels after DSC reheating was lower than that of gelatinized starch, providing evidence to support the conclusion from the DSC data that there was further melting of starch crystallites remaining in retrograded starch gels.

**Figure 5:**
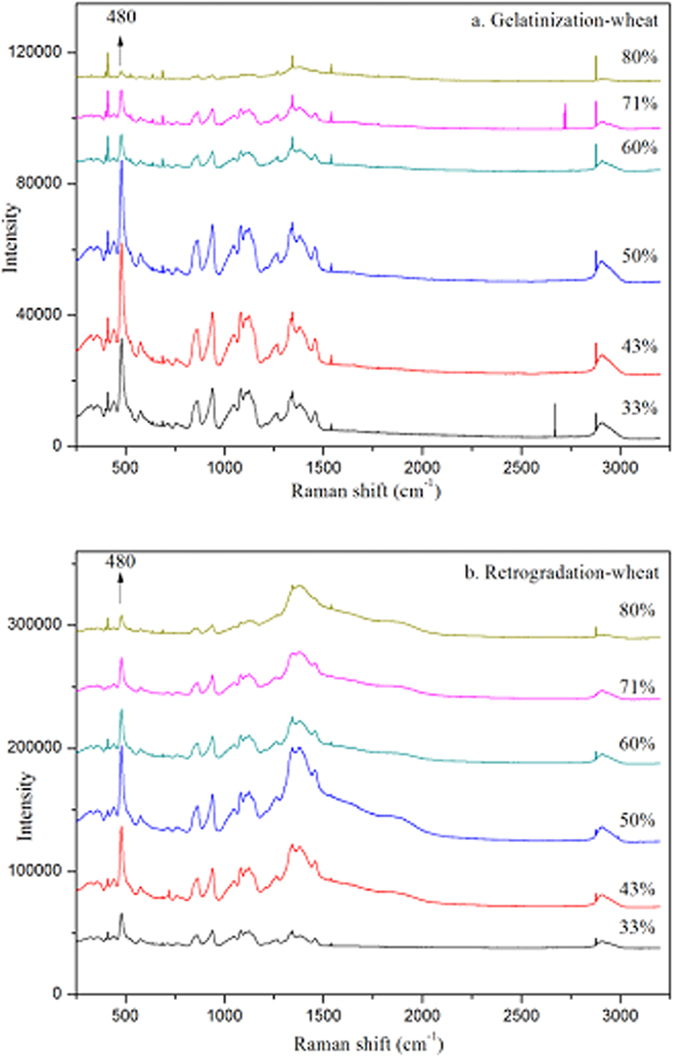
LCM-Raman spectra of gelatinized WS after DSC heating (**a**), reheated retrograded WS (**b**).

As the LCM-Raman spectra of starch samples were similar, only those of wheat starch are presented (Fig. 5). Several clear bands can be seen at 480, 865, 943, 1264 and 2900 cm^−1^, which are related to δ (CH_2_), νs (C1-O-C4), νs (C1-O-C5), skeletal (C-C-O), and ν (C-H) modes, respectively^29^,^30^,^31^. Of these bands, the ones at 480 and 2900 cm^−1^ are often used to characterize the molecular order of native starch granules or the changes in molecular order of starch samples during gelatinization or retrogradation^6^,^29^,^32^,^33^,^34^. Full width at half maximum height (FWHM) of the strong band at 480 cm^−1^ is often used to characterize the relative crystallinity of starch samples; this parameter is most responsive to changes in crystallinity^29^. Values for FWHM_R_ were higher than those for FWHM_G_ over the whole range of water content (although the difference was small at high water content), indicating that the relative crystallinity of retrograded starch gels after DSC reheating was lower than that of gelatinized starch samples, consistent with the ATR-FTIR results.

The maximum enthalpy change of retrograded starch gels was observed at water content around 50%, which does not correspond to the maximum degree of starch retrogradation. The crystallites formed during retrogradation are less ordered and less stable than crystallites in native starch granules. The observed higher enthalpy change of retrograded starch gels at low water content indicated that the enthalpy change of retrograded starch gels involves not only the melting of new starch crystallites formed on storage, but also further melting of starch crystallites remaining after gelatinization. This conclusion was supported by the ATR-FTIR and Raman spectroscopy analysis of molecular order of starch after gelatinization and after reheating of retrograded starch gels. Several studies investigated the effect of water content on retrogradation of starch^21^,^22^,^23^,^35^,^36^,^37^, but none reported an apparent value greater than 100% for the degree of retrogradation. The effect of water content on starch retrogradation, as determined by measuring DSC enthalpy change of recrystallized amylopectin, displayed a parabolic shape, with maximum retrogradation occurring in starch gels at 40–45% water content^21^,^22^,^37^.

Another interesting finding from the present study is the decreasing T_p_ and T_c_ of retrograded starch gels with increasing water content. Two hypotheses, not mutually exclusive, are proposed to interpret this observation. The crystallites formed during retrogradation could become progressively less stable with increasing water content, leading to lower T_p_ and T_c_ of retrograded starch gels. Another explanation is that the higher T_p_ and T_c_ of retrograded starch gels at low water content is due to further melting of residual starch crystallites that remained in retrograded starch gels. The melting of starch crystallites is not complete at the end of DSC heating, especially at low water content^38^,^39^. This interpretation is supported by the observation that the T_c_ of retrograded rice and wheat starch gels was slightly lower than those of native starch granules at low water content, and that the transition temperature range of retrograded starch gels decreased with increasing water content (Table 2). Taken together, we can conclude that the endothermic transition of retrograded starch gels at low water content does not reflect accurately the retrogradation behavior of gelatinized starch.

It is also interesting to note that the enthalpy change of starch gelatinization remained essentially unchanged at a water content above 71%, whereas the enthalpy change of starch retrogradation decreased progressively up to a water content of 80%. This result indicated that although the enthalpy change reached a maximum value, presumably corresponding to the complete gelatinization of starch, the retrogradation behavior of fully gelatinized starch was different at different water contents. The high water content brought about low degree of retrogradation, indicating that with increasing water content, the coming together and realignment of dispersed starch chains becomes progressively more difficult.

## Conclusions

Differential scanning calorimetry studies over a wide range of water content have shown that water strongly influences retrogradation behavior of rice and wheat starches. The DSC thermal transition parameters have demonstrated for the first time that the enthalpy change of retrograded starch gels was greater than that of native starch at water content of 33 to 50%. In combination with ATR-FTIR and Raman results, we conclude that the DSC endothermic transition of retrograded starch gels at low water content does not truly reflect the retrogradation behavior of gelatinized starch. The enthalpy change of retrograded starch gels at low water contents represents the melting of starch crystallites formed by retrogradation and of residual crystallites remaining after gelatinization. At high water content, when complete gelatinization of starch may occur, the degree of retrogradation is influenced by water content, whereas at very low and very high water content, the DSC enthalpy change indicated that little retrogradation of starch occurred.

## Additional Information

**How to cite this article**: Wang, S. *et al.* Retrogradation enthalpy does not always reflect the retrogradation behavior of gelatinized starch. *Sci. Rep.* **6**, 20965; doi: 10.1038/srep20965 (2016).

## Figures and Tables

**Table 1:** Basic composition of wheat and rice starches.

Starch	Amylose (%)	DS (%)	Water (%)	Protein (%)	Lipid (%)	Ash (%)
Rice	11.5 ± 0.3	0.89 ± 0.07	11.72 ± 0.07	0.29 ± 0.04	0.07 ± 0.01	0.24 ± 0.09
Wheat	25.0 ± 0.6	1.30 ± 0.02	10.62 ± 0.21	0.36 ± 0.02	0.11 ± 0.01	0.15 ± 0.04

Values are means ± SD. DS: damaged starch.

**Table 2:** Thermal transition temperatures of rice and wheat starch samples.

Temperatures	Water content/%	Rice starch		Wheat starch	
		G/^o^C	R/ ^o^C	G/ ^o^C	R/ ^o^C
T_o_	33	61.4 ± 0.6ab	44.1 ± 0.8a	55.1 ± 0.3a	42.8 ± 1.3ab
	43	61.2 ± 0.3a	43.5 ± 1.1a	55.2 ± 0.5a	41.9 ± 0.4a
	50	61.2 ± 0.2a	42.9 ± 0.6a	55.3 ± 1.3a	43.1 ± 0.7ab
	60	61.8 ± 0.1abc	44.2 ± 0.2a	55.5 ± 0.1a	43.8 ± 1.9ab
	71	62.1 ± 0.5c	44.2 ± 1.1a	56.0 ± 0.2a	43.1 ± 2.1ab
	80	62.0 ± 0.3bc	nd	56.1 ± 0.4a	45.4 ± 0.9b
T_p_	33	67.1 ± 0.7a	56.8 ± 0.5ab	61.0 ± 0.4a	58.3 ± 1.9c
	43	67.3 ± 0.4a	58.2 ± 0.6b	60.8 ± 0.3a	57.6 ± 1.6bc
	50	67.2 ± 0.3a	55.2 ± 0.9a	61.0 ± 0.2a	54.7 ± 1.5ab
	60	66.9 ± 0.2a	53.9 ± 0.3a	61.1 ± 0.16a	55.6 ± 1.8abc
	71	67.6 ± 0.4ab	54.7 ± 2.8a	61.8 ± 0.3b	53.6 ± 0.8a
	80	68.3 ± 0.1b	nd	62.1 ± 0.0b	54.3 ± 0.1a
T_c_	33	71.3 ± 0.7a	67.8 ± 1.8bc	64.6 ± 0.3b	65.3 ± 0.2d
	43	71.0 ± 0.4a	68.9 ± 2.0c	64.8 ± 0.2ab	64.8 ± 0.2cd
	50	71.3 ± 0.3a	65.9 ± 0.8b	65.6 ± 0.4b	64.5 ± 0.2cd
	60	71.8 ± 0.6a	63.2 ± 0.4a	66.2 ± 0.2b	64.1 ± 0.9bc
	71	73.9 ± 0.1b	62.5 ± 0.2a	67.0 ± 0.2c	63.4 ± 0.8ab
	80	73.3 ± 0.3b	nd	67.4 ± 0.4c	62.7 ± 0.1a
T_c_–T_o_	33	9.9 ± 0.1a	23.2 ± 1.4b	9.5 ± 0.5a	22.5 ± 1.1b
	43	9.8 ± 0.1a	25.4 ± 2.2b	9.6 ± 0.6a	22.9 ± 0.2b
	50	10.1 ± 0.4a	23.0 ± 1.0b	10.3 ± 1.2a	21.4 ± 0.6b
	60	10.0 ± 0.5a	19.0 ± 0.5a	10.7 ± 0.3ab	20.3 ± 2.2b
	71	11.7 ± 0.6b	18.3 ± 0.9a	11.0 ± 0.2ab	20.3 ± 1.3b
	80	11.3 ± 0.6b	nd	11.3 ± 0.3b	17.3 ± 0.9a

Values are means ± SD.Values with the same letters in the same column for each starch are not significantly different (p < 0.05).

G: gelatinization; R: retrogradation

nd: not determined.

**Table 3:** Enthalpy change of native and retrograded starches.

Starch	Water content/%	ΔH_G_J/g	^a^ΔH_R_ J/g	^b^ΔH_R_ J/g	DR%
Rice starch	33	2.6 ± 0.1a	3.9 ± 0.0c	4.2 ± 0.2a	153.1 ± 5.7f
	43	4.6 ± 0.1b	5.6 ± 0.2e	5.2 ± 0.4b	121.9 ± 3.4e
	50	5.5 ± 0.1c	7.0 ± 0.4f	6.6 ± 0.0b	82.8 ± 5.0d
	60	14.0 ± 0.2d	4.7 ± 0.4d	4.6 ± 0.1a	33.3 ± 2.7c
	71	14.4 ± 0.7d	1.4 ± 0.2b	nm	9.9 ± 0.6b
	80	14.3 ± 0.2d	0a	nm	0a
Wheat starch	33	2.9 ± 0.0a	6.2 ± 0.2e	4.4 ± 0.5b	214.8 ± 5.0e
	43	4.1 ± 0.1b	6.0 ± 0.0e	4.6 ± 0.7b	146.1 ± 2.5d
	50	5.5 ± 0.0c	5.5 ± 0.1d	4.1 ± 0.3b	100.1 ± 2.3c
	60	8.5 ± 0.1d	2.6 ± 0.4c	3.3 ± 0.1a	30.5 ± 4.4b
	71	10.7 ± 0.4e	1.8 ± 0.1b	nm	16.4 ± 0.3a
	80	10.7 ± 1.1e	0.5 ± 0.0a	nm	4.7 ± 0.3a

Values are means ± SD. Values with the same letters in the same column for each starch are not significantly different (p < 0.05).

a: reheating retrograded starch gels; b: reheating retrograded starch powders/water mixtures; DR% was calculated based on the enthalpy change of regrograded starch gels.

nm: not measured.

**Table 4:** The ratios of 1047/1022 cm^−1^ and FWHMs of the band at 480 cm^−1^ of starch samples.

Starch	Water content/%	1047/1022cm^−1^_G_	1047/1022cm^−1^_R_	FWHM_G_	FWHM_R_
Rice starch	33	0.695 ± 0.017c	0.649 ± 0.023c	15.47 ± 0.08a	15.54 ± 0.09a
	43	0.647 ± 0.008b	0.625 ± 0.007abc	15.45 ± 0.54a	16.38 ± 0.44b
	50	0.657 ± 0.006b	0.635 ± 0.005bc	15.59 ± 0.05a	15.84 ± 0.36ab
	60	0.646 ± 0.009b	0.645 ± 0.007c	15.73 ± 0.01a	15.77 ± 0.07ab
	71	0.595 ± 0.014a	0.595 ± 0.032ab	15.31 ± 0.09a	15.36 ± 0.58a
	80	0.604 ± 0.012a	0.586 ± 0.011a	15.66 ± 0.23a	16.38 ± 0.25ab
Wheat starch	33	0.694 ± 0.015b	0.652 ± 0.006b	15.94 ± 0.35ab	16.32 ± 0.08b
	43	0.687 ± 0.010b	0.655 ± 0.007b	16.53 ± 0.02b	16.99 ± 0.24c
	50	0.680 ± 0.001b	0.664 ± 0.008b	15.92 ± 0.04ab	16.23 ± 0.24b
	60	0.689 ± 0.017b	0.660 ± 0.016b	15.67 ± 0.35a	15.78 ± 0.25a
	71	0.662 ± 0.014a	0.658 ± 0.014b	16.32 ± 0.41b	16.40 ± 0.04b
	80	0.647 ± 0.037a	0.623 ± 0.005a	15.53 ± 0.50a	16.44 ± 0.28b

Values are means ± SD. Values with the same letters in the same column for each starch are not significantly different (p < 0.05).

G represents native starch after DSC heating; R represents retrograded starch gels after DSC reheating.
